# Oral pathology and overweight among pupils in government primary schools in Cameroon: a cross-sectional study

**DOI:** 10.1186/s12903-023-02941-z

**Published:** 2023-05-12

**Authors:** Lionel Berthold Keubou Boukeng, Claude Axel Minkandi, Leonie Nzefa Dapi

**Affiliations:** 1 Department of Public Health, Faculty of Medicine and Biomedical Sciences, University of Yaounde I, P.O. Box 1364, Yaoundé, Cameroon; 2 Department of Medicine and Optometry, Inst of Health and Life Sciences, Linnaeus University, P.O. Box 39182, Kalmar, Sweden

**Keywords:** Overweight, Tooth decay, Gingivitis, Periodontitis, Pupils, Cameroon

## Abstract

**Background:**

Tooth decay and periodontal diseases are the main oral pathologies in the world. The prevalence of overweight in children has increased worldwide. Overweight children have alterations in the composition of saliva and excessive consumption of saturated fatty acids tend to slow the metabolism of carbohydrates in the oral cavity leading to tooth decay, periodontal disease and others oral disorders. The aim of this study was to assess the relationship between oral pathologies and overweight in pupils of primary schools of Cameroon.

**Methods:**

A cross-sectional study was carried out from June to August 2020 in four government primary schools selected through cluster sampling in Yaounde. 650 pupils aged between 6 and 11 years were enrolled. Data collected included anthropometric, oral pathologies, quality of oral hygiene and feeding habits. Data were analysed with the SPSS 26.0 statistical software and binary logistic regression was used to determine the risks of oral pathologies in overweight pupils. P-value of 0.05 was considered statistically significant.

**Results:**

The prevalence of overweight was 27% (95% CI: 23.5—30.5). The main oral pathologies was tooth decay (60.3%). Binary logistic regression revealed that overweight pupils were significantly 1.5 times more likely to develop tooth decay than non-overweight pupils (95% CI: 1.1—2.4).

**Conclusion:**

Overweight, tooth decay are prevalent among pupils. Overweight pupils have a higher risk of developing tooth decay compared to non-obese pupils. An integrated package of oral and nutritional health promotion activities is necessary in primary schools in Cameroon.

## Background

Tooth decay and periodontal diseases are the main oral pathologies seen worldwide with 60–90% of pupils affected [1–3]. In Africa, 38–45% of children are affected and the prevalence of 43% and 75% for tooth decay and periodontal disease respectively were reported in Cameroon by a study conducted in urban and semiurban areas of the Mfoundi division among children between 6 and 12 years old in 2015 [4]. Also, the prevalence of overweight in children has increased in all parts of the world [5]. Projections in the Africa indicated a prevalence of 12.7% in 2020 [6]. Studies carried out in Cameroon reported a prevalence between 12.5% and 18.8% among pupils [7, 8]. Several factors are associated with the burden of oral pathologies and overweight in children including feeding habits characterized by nutritional transition with excessive consumption of foods rich in refined carbohydrates and saturated fatty acid [1]. Overweight children have alterations in the composition of saliva due to high level of sugar that has been linked to a higher incidence of cariogenic microorganisms leading to tooth decay, periodontal disease, loss of dental organs and other oral disorders such xerostomia and malocclusions [1]. Similarly, in children with a higher Body Mass Index (BMI), excessive consumption of saturated fatty acids tends to slow the metabolism of carbohydrates in the oral cavity enabling alterations in the composition of saliva as favourable factors for conditioning those diseases [1]. Oral pathologies and overweight can lead to complications such as cardiovascular and respiratory diseases, type II diabetes which require treatment in specialized services and follow-up to avoid reoccurrence specially in children [1]. In Cameroon, the primary school enrolment rate of children aged 6 to 11 years was 84% in 2018 [7, 9]. This age constitutes an opportunity in the human life cycle for health promotion in order to reduce the burden of diseases [10]. Furthermore, the demographic load of pupils represents a key target for the implementation of prevention and promotional activities of nutritional and oral health [7]. The aim of this study is to assess the relationship between oral pathologies and overweight in pupils in Cameroon.

## Methods

### Study design and setting

A cross sectional study was conducted in Yaounde, capital city of Cameroon. All the socio-economic groups and ethnic groups of the country are found in Yaoundé city as well as the primary cycle of our educational system [11]. In Cameroon, primary education is compulsory and government primary schools are free of charge, thus resulting to higher number of pupils Our study was conducted from 1 June to 31 August 2020. Pupils aged between 6 and 11 years, present at the time of the survey and whose parents gave their consent were included in the study. Pupils with chronic conditions which could have an impact on food intake and alter their metabolism, as well as those with signs of puberty because of rapid growth spurts at this stage of life that could alter anthropometric measurements, and those whose parents wanted them to withdraw from study for any reason were excluded.

### Sampling

The pupils were recruited through the sampling cluster method. The process began with the writing of the names of all the 34 schools on a non-transparent paper after which all the papers were placed in a box. Then, a third party was asked to randomly pick four papers. Four government primary schools were chosen and all the pupils present at the time of the survey were enrolled in our study. The sample size was calculated using the following formula $n={k\left(\frac{\mathrm{z}1-\mathrm{\alpha }/2 \sqrt{\mathrm{P}\left(1-\mathrm{P}\right)}}{\mathrm{d}}\right)}^{2}$ with a margin of error of 5%, z1-α / 2 = 1.96, a cluster effect of 1.5, a precision of 5% and a prevalence of tooth decay of 43% reported by Trinita and al [4, 7]. The size was adjusted to take into account withdrawals and refusals estimated at 10%. A size of 628 was obtained.

### Measurements and questionnaires

Anthropometric measurement, dental examination and feeding habits were handled by trained staff using standardized procedures and instruments. Parents were also asked to fill the first part of the questionnaire on socio-demographic information. The socio-demographic data from the guardian questionnaire included information on gender (male or female) and date of birth of the pupils, the educational level and occupation of the mother and socioeconomic status of the family. The educational level of the mother was categorized into primary (primary school), secondary (college and vocational schools), and higher (university level or higher) levels. The guardian’s occupation was categorized as student, unemployed, formal sector and informal sector worker. The socio-economic status was determined using the classification system from the National Institute of Statistics of Cameroon [8, 11]. In addition, self-reported age, height and weight for each mother was obtained, from which maternal Body Mass Index (BMI) was calculated.

### Anthropometric measurements

Concerning anthropometric data, body weight (kg) was measured to the closest 0.1 kg using an electronic weighing scale (OMRON BF 511 for body composition monitor). Pupils weight were taken in the morning while dressed in light clothes and without shoes [12, 13]. Standing height (cm) was measured to the closest 0.1 cm using a portable stadiometer (SECA model 206) made from a tape measure fixed to the wall and headpiece equipped with a level to ensure a right angle ([14]). Measurements were taken twice and in case of discrepancy between the first two, a third measure was taken and considered as the value to be recorded. From these measurements, BMI (kg/m^2^) was calculated and BMI standard deviations scores (BMI z-score) were derived using the age (calculated by subtracting the date of birth from the date of examination) and sex specific WHO growth references for children aged 5–19 [6]. In this study, the term overweight concerned all pupils with a BMI z-score ≥  + 1.0.

### Dental examination

The oral cavity examination was performed in a ventilated room with a single-use tray made of dental plane mirror, probe No.17, dental precell, a World Health Organization periodontal probe, and a head torch. It was done using the Australian Oral Health Assessment Tool and Chicago classification of periodontal diseases adopted in 2017 [15, 16]. For dental tissues, the investigator used the probe to search for carious cavities on all the surfaces of each tooth. In the maxilla, they stood behind the patient in a 12 o’clock position and used a mirror; in the mandible, they examined the teeth in direct vision in a position between 6 and 9 o’clock. A tooth was considered to be affected by caries if it resisted and/or clung to the probe accompanied by signs such as colour change or the presence of white spots on the faces. The investigator reported decayed, filled, and missing teeth for permanent denture and only decayed and filled teeth for temporary denture. The examination concerned the soft tissues; the diagnosis of gingivitis was made when the inflammation was limited to the gum tissue and only caused by bacterial biofilm. Also, periodontitis was made using one of the three criteria of the 2017 Chicago classification: presence of a periodontal pocket of at least one millimetre [16]. Oral hygiene classification in three groups was also done using the criteria of Australian tool (15).

### Feeding habits

Data on feeding habits was collected using multiple 24-h dietary recall method on three non-consecutive days including the weekend [17]. The method was applied on Monday, Tuesday and Thursday to find out the quantities of food and drinks consumed on Sunday, Monday and Wednesday respectively. The evaluators showed the students a catalog in which the majority of our meals were presented in different packaging and calibrated at 1 g, 5 g, 10 g, 15 g, 25 g, 50 g, 100 g, 200 g for foods as well as 5 ml, 10 ml and 20 ml for beverages and snacks. Then, they were asked to point out all the meals they had eaten on the selected days. All the selected packages were entered into the Nutrition Maker Plus 3.0.52.10 software in order to have their micro and macronutrient contents.

### Statistical analysis

Data was analysed with Nutrition Maker Plus 3.0.52.10 software for the food component, AnthroPlus (Macro SPSS, WHO) for the anthropometric component and SPSS 26.0 statistical software (SPSS Inc., Chicago, IL) for the overall analysis. The qualitative variables were presented with their number and percentage. Kolmogorov–Smirnov test was used for quantitative variables. Those with a normal distribution were summarized with the mean and standard deviation while the median and interquartile range were used for variables presenting any other distribution. Oral pathologies represented our outcomes and were transformed into a binary scale. Binary logistic regression was used to control confounding factors such their eating habits as well as socio-demographic and anthropometric status of their guardians. Only variables with a significant p-value or close to significance were included in the regression model and a p-value less than 0.05 was considered statistically significant.

## Results

### Sociodemographic characteristics of pupils and their guardians

The median age of pupils was 10.1 (9.6–11.4) years; it consisted of 352 girls (54.2%) and 298 boys (45.8%). Two hundreds and seventy-nine pupils (42.9%) belonged to the low socio-economic level. Three hundred and fifty (53.8%) guardians of pupils had reached secondary school and 504 (77.5%) worked in the informal sector (Table 1).

**Table 1:** Characteristics of guardians’ pupils in Yaounde (*N* = 650)

Guardians pupils characteristics	Frequency (N)	Percentage (%)
**School level**		
Primary	156	24.0
Secondary	350	53.8
Higher	144	22.2
**Age (in years)**		
< 35	189	29.1
≥ 35	461	70.9
**Body Mass Index (kg/m**^**2**^**)**		
**< **25	230	35.4
≥ 25	420	64.6
**Occupation**		
Formal sector worker	146	22.5
Informal sector worker	504	77.5

### Feeding habits of pupils

A daily frequency of three meals was observed in 413 pupils (63.5%). Their median consumption of carbohydrates, lipids, proteins was 280.1 (115.5–530) g/day, 24 (19.1–40.1) g/day, 39.9 (34.1–69) g/day respectively; that of calcium was 430.9 (358.1–1365) mg/day. The mean fluoride consumption was 1.51 ± 0.5 mg/day (Table 2).

**Table 2:** Daily macronutrient consumption of pupils in Yaounde (*N* = 650)

Daily macronutrients consumption	Frequency (N)	Percentage (%)
**Frequency of meals (number per day)**		
< 2	222	34.2
≥ 2	428	65.8
**Calcium (mg/day)**		
< 1300	474	72.9
≥ 1300	176	27.1
**Fluor (mg/day)**		
< 1	321	49.4
≥ 1	329	50.6
**Carbohydrates (g/day)**		
< 130	208	32.0
≥ 130	442	68.0

Recommended Daily Allowances (RDA): 1000 mg for calcium; 1 mg for fluor; 130 g for carbohydrates

### Oral hygiene of pupils

Oral hygiene examination revealed 202 pupils (31.1%) with plaque in several places in the mouth or severe halitosis, 192 (29,5%) with plaque in 1–2 areas of the mouth or halitosis and 256 (39.4%) had a clean mouth.

### Weight categories of pupils

Values ​​of the Body Mass Index specific to the age and sex of pupils showed a deviation of their z-score to the right from the WHO growth standards. Weight status was normal in 427 pupils (65.7%). Overweight pupils were found in 175 pupils (26.9%) with a 95% confidence interval ranging from 23.5 to 30.5 (Table 3).

**Table 3:** Nutritional status of pupils in Yaounde (*N* = 650)

Nutritional status	Frequency (N)	Percentage (%)	95% Confidence Interval
Stunting (-1 ≤ BMI z-score)	48	7.4	[5.5; 9.7]
Normal (-1 < BMI z-score < + 1)	427	65.7	[61.9; 69.3]
Overweight (BMI z-score ≥ + 1)	175	27	[22,2; 32,2]

### Oral pathologies among pupils

Tooth decay was the most common oral pathology with 60.7%. Periodontal disease was found in 346 pupils (53.2%); of these, gingivitis was present in 33.1% and periodontitis in 20.1% (Table 4).

**Table 4:** Oral pathologies of pupils in Yaounde (*N* = 650)

Oral pathologies	Frequency (n)	Percentage (%)	95% Confidence Interval
**Tooth decay**	392	60.3	[56.4; 64.1]
**Periodontal diseases**	346	53.2	[49.3; 57.1]
Gingivitis	215	33.1	[27.2; 37.8]
Parodontitis	131	20.1	[16.1;24.4]
**Cheilitis and lip ulcerations**	26	4.0	[2.6;5.8]
**Glossitis**	15	2.3	[1.3; 3.8]
**Hyposialia**	17	2.6	[1.5;4.2]
**Traumatic lesions of the tooth**	67	10.3	[8.1;12.9]

### Relationship between tooth decay and obesity

Binary logistic regression revealed that overweight pupils were significantly 1.5 times more likely to have tooth decay after adjusting for their age, carbohydrate consumption and oral hygiene than the non- overweight pupils with an adjusted p value of 0.011 (Table 5).

**Table 5:** Relationship between tooth decay and overweight among pupils in Yaounde (*N* = 650)

Pupils ‘characteristics	Tooth decay		Adjusted Odds Ratio (95% CI)	Adjusted *p*-value (95% CI)
	**Yes (*n* = 392)**	**No(*n* = 298)**		
**Overweight (BMI z-score ≥ + 1)**	110 (62.9%)	65 (37.1%)	1.5 [1.09;2.35]	0.011*
**Age of pupil** (< 10,11 years)	211 (59.4%)	144 (40.6%)	0.305 [0.18;0.50]	0.01*
**Gender** (Male)	184 (56.1%)	144 (43.9%)	1,30 [0.88;1.92]	0.18
**Guardian ‘age** (< 35 years)	126 (66.7%)	66 (33.3%)	1,0 [0.64;1.56]	0.98
**Guardian Occupation** (Informal Sector Worker)	114 (67.5%)	55 (32.5%)	1.13 [0.71;1.81]	0.59
**Calcium** (> 1300 mg/day)	126 (50%)	126 (50%)	0.86 [0.50;1.47]	0.56
**Fluor** (< 1 mg/day)	187(58.3%)	134(41,7%)	0,91 [0.56;1.47]	0.03*
**Carbohydrates** (< 130 g/day)	130 (62.5%)	78 (37.5%)	1,19 [0.75;1,88]	0.045*
**Oral Hygiene** (bad)	113 (55.9%)	89 (44.1%)	2,41 [1.46;3.97]	0.001*

^*^: Significance level of 0.05

## Discussion

Our sample consisted of 352 (54.2%) female and 298 (45.8%) male pupils. Our results are closer to those of Agbor et al. who reported a female predominance (54.9%) in their series conducted in primary schools in urban and rural areas of the West Region in 2018 [18]. However, they differ from that of Lifoter et al. who found 51.5% of male pupils in their study in urban and rural primary schools in the North West Region in 2014 [8]. These findings indicate that there is no gender discrimination in the admission into our primary education system (level). The lower socio-economic class was the most represented with 42.9%, followed by the middle class,30.8%. These findings are similar to those of Lifoter et al. who found 43.1% and 25.85% respectively [8]. However, they contrast with those of Choukem et al. who found the upper class to be the most represented in their study conducted on children aged 3 to 13 years in the city of Douala in 2017 [7]. In this study, the authors grouped the middle and upper classes together as one entity in their methodology. Moreover, government schools are accessible to all social strata in the city of Yaounde. In our study, the average age of mothers of the pupils was 38.47 (± 5.61) years. These data are similar to those of the study conducted by Choukem et al. with 35(± 6) years [7]. Furthermore, 53.8% of these mothers had reached secondary school in our education system and 77.5% of them were working in the informal sector. These results are closer to those of Ntombizodwa et al. who found 86% and 50% respectively in their study in grade 6 and 7 pupils in the Tshwane Health District in the western part of Pretoria, South Africa in 2017 [19]. This difference in percentage could be explained by the choice of study site. This is because the economic opportunities in the two cities are different; Pretoria is a growing city with fluorescent job opportunities and market.

The prevalence of overweight was 27%. This result is higher than those reported by many studies in the sub-Saharan region that also used the 2007 WHO references: 12.5% in the study by Choukem et al. in Douala [7]; 18.8% for Lifoter et al. in Bamenda (8); 12% in the study by Ntombizodwa et al. in the suburbs of Pretoria [19]; and 11% according to Ahmed et al. in pupils of primary schools in Khartoum, Sudan in 2013 [20]. However, the trend is more alarming in developed countries: 36.7% in the study by Kennedy et al. conducted in children in Manotiba State Canada in 2017 [21]. Data from this survey, in accordance with WHO predictions, indicate a worsening in our environment and several hypotheses have been proposed in the related literature to explain this phenomenon, particularly the nutritional transition. In developing countries such as ours, there is a change in eating habits, with the adoption of a diet that is increasingly rich in carbohydrates and fats; this trend also affects school-age children in their families and schools.

Tooth decay is the most common oral pathology with 392 cases (60.7%). Our results differ from those of Agbor et al. who reported 28.1% [18] as well as Trinita et al. who found 43% in pupilsfrom 6 to 12 years old in the Mfoundi Division in 2015 [4]. However, they are consistent with WHO data which estimate that 45–55% of school-age children are affected in Africa [2]. This prevalence can be explained by the consumption of substances rich in carbohydrates and especially refined sugar; they constitute substrates for bacteria of the oral flora which, by metabolism, produce acids that cause tooth decay. The second factor at the genesis of tooth decay is oral hygiene. Failure to practice oral hygiene, favours the installation of a pathogenic flora that leads to the tooth decay process [1]. Periodontal diseases were the second group of oral pathologies with 53.2%. Our results are lower than those of Trinita et al. and Hubert et al. who reported frequencies of 75% and 77.7% in their studies conducted in the Mfoundi Division and in Yaounde respectively in 2015 and 2016  [4, 22]. This could be explained by the choice of our study sites. Indeed, Trinita et al. included rural areas in their study where the respect of oral hygiene rules is not part of the habits. In these areas, pupils do not respect these rules due to a lack of means to acquire the necessary kit to maintain good hygiene and also because they do not know about these rules. However, Hubert et al. studied children with disabilities, which reduces their autonomy in controlling oral hygiene [4, 18]. The non-respect of hygiene rules favours the formation of bacterial plaque, which causes inflammation of the tissues that support dental germs; the process starts with gingivitis and is called periodontitis in the case of loss of gum tissue attachment [1, 4]. According to the literature, gingivitis was the most prevalent lesion followed by periodontitis with 215 (53.2%) and 131 (37.87%) cases respectively [4].

, In our study, overweight pupils were 1.5 times more likely to develop tooth decay than non-overweight pupils after adjusting their age, daily fluoride and carbohydrate intake. This result , differs from those reported by Ahmed et al. who found a significant negative association between dental index and body mass index in a study conducted in Sudan in 2013 on 360 children [20]. similarly, Ntombizodwa et al. in South Africa, in a study conducted in public schools in 2016 reported that there was no correlation between the proportion of decayed teeth and body mass index [19]. However, this is identical to Adeniyi et al. who also found a risk of 1.5 in their study of 973 children in government and private schools in Lagos in 2015 [23]. Indeed, tooth decay and overweight are both multifactorial entities with a genetic predisposition as well as an environmental influence. The school environment is characterised by an abundance of shops selling products rich in carbohydrates such as lollipops, ice cream, pastries, confectionery, chocolate, caramel, sugary drinks, doughnuts [12, 24]; these products are accessible and abundantly consumed by overweight pupil. The hydrates contained in these products are mostly slow-metabolizing sugars, particularly sucrose, which are ideal substrates for the bacteria of the oral flora, i.e., Staphyloccus aerus and Streptococcus mutans [4]. These bacteria degrade them into lactic acid, which causes the "white spots" observed on the enamel of dental germs and represents the starting point of tooth decay. In addition, their family environment is also marked by the nutritional transition observed in our milieu. This is materialised by the provision of foodstuffs such as sweets, pastries, ice cream, lemonades and sweetened drinks, which are highly cariogenic, by their parents and/or guardians [12, 25]. These overweight pupils develop appetite and addiction to these products, thus creating a vicious circle that maintains and aggravates this co-mordibidity due to their over-consumption.

## Conclusion

Overweight, tooth decay, gingivitis and periodontitis are prevalent among pupils in Cameroon. Overweight pupils have a higher risk of developing tooth decay compared to non-obese pupils. An integrated package of oral and nutritional health promotion activities is needed in primary schools in Cameroon.

## Data Availability

Data sharing: participant level data is available from the first author (corresponding author) on reasonable request.

## Abbreviations

CI: Confidence interval
BMI: Body mass index
RDA: Recommended daily allowances
SPSS: Statistical package of social sciences
WHO: World health organization
